# Metabolomic Analysis of Aqueous Humor Identifies Aberrant Amino Acid and Fatty Acid Metabolism in Vogt-Koyanagi-Harada and Behcet’s Disease

**DOI:** 10.3389/fimmu.2021.587393

**Published:** 2021-02-22

**Authors:** Jing Xu, Guannan Su, Xinyue Huang, Rui Chang, Zhijun Chen, Zi Ye, Qingfeng Cao, Aize Kijlstra, Peizeng Yang

**Affiliations:** ^1^The First Affiliated Hospital of Chongqing Medical University, Chongqing Branch of National Clinical Research Center for Ocular Diseases, Chongqing, China; ^2^University Eye Clinic Maastricht, Maastricht, Netherlands

**Keywords:** Vogt-Koyanagi-Harada disease, Behcet’s disease, metabolomics, pathway, amino acids, fatty acids

## Abstract

To investigate aqueous metabolic profiles in Vogt-Koyanagi-Harada (VKH) and Behcet’s disease (BD), we applied ultra-high-performance liquid chromatography equipped with quadrupole time-of flight mass spectrometry in aqueous humor samples collected from these patients and controls. Metabolite levels in these three groups were analyzed by univariate logistic regression. The differential metabolites were subjected to subsequent pathway analysis by MetaboAnalyst. The results showed that both partial-least squares discrimination analysis and hierarchical clustering analysis showed specific aqueous metabolite profiles when comparing VKH, BD, and controls. There were 28 differential metabolites in VKH compared to controls and 29 differential metabolites in BD compared to controls. Amino acids and fatty acids were the two most abundant categories of differential metabolites. Furthermore, pathway enrichment analysis identified several perturbed pathways, including pantothenate and CoA biosynthesis when comparing VKH with the control group, and D-arginine and D-ornithine metabolism and phenylalanine metabolism when comparing BD with the control group. Aminoacyl-tRNA biosynthesis was altered in both VKH and BD when compared to controls. Our findings suggest that amino acids metabolism as well as two fatty acids, palmitic acid and oleic acid, may be involved in the pathogenesis of BD and VKH.

## Introduction

Uveitis known as intraocular inflammation is one of the leading causes of visual impairment and blindness in the world (1, 2). Vogt-Koyanagi-Harada (VKH) disease and Behcet’s disease (BD) are two of the most commonly seen uveitis entities in China (3, 4). The incidence rate of uveitis in China is 111.3 persons per 100,000 person-years (5) and a frequency of 12.7% and 8.7% has been reported for VKH and BD in Chinese uveitis patients, respectively (4). VKH disease is a multisystemic autoimmune disease characterized by recurrent bilateral granulomatous panuveitis accompanied with systemic involvements including poliosis, alopecia, vitiligo, central nervous system, and auditory abnormalities (6). BD is recognized as an autoinflammatory disorder accompanied with recurrent uveitis, oral aphthae, genital ulcers, and typical skin lesions (7). The exact pathogenesis of both VKH disease and BD remains unclear and omics analysis may advance the understanding of these diseases.

Metabolomics is a rapidly expanding field in life science which leads to the growing interest in metabolism (8). Metabolites, serving as downstream products of transcription, translation, and post-translational protein modification, can reflect and be influenced by local physiological events (9). Therefore, a study of metabolomics could contribute to a better understanding of the pathophysiology of intraocular inflammation. Recent metabolomic studies in VKH disease or BD, using sweat or urine samples have revealed a specific metabolite profile (10, 11). Aqueous humor fills the anterior chamber of the eye and contributes to a normal intraocular homeostatic environment (12). Its composition may probably be a better reflection of local physiological changes associated with intraocular diseases than the analysis of samples obtained from peripheral sites. As far as we know, no aqueous metabolomic study has been reported for VKH or BD and this was therefore the subject of the study described here.

Liquid chromatography/mass spectrometry (LC/MS) is a commonly applied technique for untargeted metabolomics detection with robust reliability and reproducibility (13–15). In view of these features, we performed an untargeted metabolomic analysis of aqueous humor samples from VKH disease and BD patients taken during cataract surgery and compared it with samples from senile cataract controls, applying ultra-high-performance liquid chromatography equipped with quadrupole time-of flight mass spectrometry (UPLC-Q-TOF/MS).

## Materials and Methods

### Participants

Individuals selected for this study included 15 VKH patients, 15 BD patients, and 15 senile cataract controls. The VKH and BD patients included, had developed cataract as a complication of their uveitis. The demographic data of the cohorts were collected (16) and shown in **Table 1**. Criteria of an international committee and those developed by our group were used to make the diagnosis of VKH disease (17, 18). Diagnosis of BD was based on the International Study Group’s criteria (19). Patients with any unclear diagnosis were excluded from the study. We also excluded patients with other ocular condition such as optic nerve disease, macular abnormalities or diabetic retinopathy. As for controls, the exclusion criteria were subjects with uveitis and a history of any other autoimmune disease.

**Table 1:** Demographic and clinical characteristics of participants.

Characteristics	VKH	BD	Controls	*p* value
Number (n)	15	15	15	–
Laterality (OD/OS)	7/8	5/10	8/7	–
Age (years)	45.8 ± 9.79	35.93 ± 10.72	60.6 ± 7.44	<0.001^a^
Gender (Male/Female)	8/7	9/6	6/9	0.54^b^
IOP (mmHg)	13.5 ± 2.8	15.7 ± 3.5	15.1 ± 2.6	0.13^a^
Systemic corticosteroids, (n, %)	15 (100%)	15 (100%)	n.a.	–
Immunosuppressant, (n, %)	15 (100%)	13 (86.67%)	n.a.	0.48^b^
Biological agents, (n, %)	0	0	n.a.	–

VKH, Vogt-Koyanagi-Harada disease; BD, Behcet’s disease; IOP, intraocular pressure; n.a., not applicable.

Continuous variables are expressed as mean ± standard deviation.

^a^Analyzed by ANOVA; ^b^Analyzed by Pearson chi-square test/Fischer’s exact test.

All participants underwent cataract surgery at the First Affiliated Hospital of Chongqing Medical University (Chongqing, China) from August, 2017 to May, 2019. Our method for cataract removal in uveitis patients has been reported elsewhere (20). Signed informed consents were obtained from all enrolled participants. The study received the approval of the Ethics Committee of the First Affiliated Hospital of Chongqing Medical University and followed the tenets of the Declaration of Helsinki.

### Sample Collection and Preparation

Anterior chamber paracentesis was performed to collect approximately 200 μl of aqueous humor samples at the beginning of the cataract surgery under the sterile condition. Care was taken to avoid contamination of samples with blood or touching intraocular tissues. The specimens were stored at −80°C until UPLC-Q-TOF/MS analysis. All specimens were thawed at 4°C. To remove protein, 80 μl aqueous humor aliquots were mixed with 320 μl of cold methanol/acetonitrile (1:1, v/v) which was then centrifuged for 20 min (14,000 g, 4°C). The supernatant was dried in a vacuum centrifuge. Quality control (QC) specimens consisting of mixing with 10 μl of each sample were analyzed with other samples to monitor the repeatability and stability of instrument. The QC specimens were inserted regularly in the cohort of tested samples, which could monitor the reliability of the whole procedure.

### UPLC-Q-TOF/MS Analysis

Aqueous metabolomic analyses were applied using an Agilent 1290 Infinity LC system (Agilent Technologies, Santa-Clara, California, USA) coupled with an AB SCIEX Triple TOF 6600 System (AB SCIEX, Framingham, MA, USA). For chromatographic separation, ACQUITY HSS T3 1.8 mm (2.1 mm × 100 mm) columns were used for sample analyses in both positive and negative modes. The column temperature was set at 25°C. The mobile phase was set as follows: A = 25 mM ammonium acetate and 25 mM ammonium hydroxide in water, and B = acetonitrile. The elution procedure was set as follows. After keeping for 0.5 min, the gradient of 95% B was linearly reduced to 65% in 6.5 min. Then it was decreased to 40% in 1 min. After keeping 40% B for 1 min, it was increased to 95% in 10 s. A 3-min re-equilibration period was employed in the procedure. The delivery flow rate was set at 300 μl/min, and 2 μl aqueous specimen was injected into the column.

UPLC-Q-TOF/MS was conducted in both positive and negative ion modes. Electrospray ionization source conditions were set as Ion Source Gas1 = 60 psi, Ion Source Gas2 = 60 psi, curtain gas = 30 psi, source temperature = 600°C, and IonSpray Voltage Floating = 5500 V (+) and −5500 V (−). The MS/MS spectra were obtained by using information dependent acquisition (IDA) coupled with the selected high sensitivity mode. The parameters were set as the collision energy = 50 eV (+) and −20 eV (−), declustering potential = 60 V (+), and −60 V (−), excluding isotopes within 4 Da, and candidate ions to monitor per cycle = 6.

### Metabolomics Data Processing

The raw UPLC-Q-TOF/MS data were converted to MzXML files using Proteo Wizard MSconverter, and then imported with XCMS online software for data processing (https://xcmsonline.scripps.edu/landing_page.php?pgcontent=mainPage). The parameters including peak picking, retention time, and peak grouping were aligned by XCMS. We completed compound identification of metabolites by comparing the accuracy m/z value (<25 ppm) and MS/MS spectra. After being normalized, the processed data were uploaded into SIMCA-P (version 14.1, Umetrics, Umea, Sweden) for multivariate data analysis. Then we conducted principal component analysis (PCA) and partial least square discriminant analysis (PLS-DA) to acquire an overview of the metabolomics data in both positive and negative models. The contribution of each metabolite was calculated from the PLS-DA model and expressed as variable importance in the projection (VIP) score. To evaluate the significance of metabolites, those metabolites with a VIP score >1 were analyzed statistically with Student’s t-test. We performed the Benjamini–Hochberg procedure for multiple testing adjustments, whereby the critical false discovery rate (FDR) was set to 0.05. The Human Metabolome Database (HMDB) (https://hmdb.ca/) was used to define the classes of metabolites.

### Bioinformatics Analysis

Venny software version 2.1.0 (https://bioinfogp.cnb.csic.es/tools/venny/) was used to generate a Venn diagram which could directly show the common and unique differential metabolites among the three groups. Multi Experiment Viewer (MeV) software version 4.9.0 was applied for the heat plot and hierarchical cluster analysis of metabolites. A Volcano plot was made with Graphpad Prism 7.0. MetaboAnalyst (https://www.metaboanalyst.ca/), an open database source, was used to identify metabolic pathways and to perform pathway enrichment analysis.

### Statistical Analysis

Statistical analysis of the data was performed using SPSS 22.0. Results are presented as the mean ± standard deviation (SD) for continuous variables. Normality was assessed by the Shapiro-Wilk test and *p* > 0.05 referred to normally distributed data. Student’s t-test, Variance (ANOVA), Fisher’s exact test and the Pearson chi-square test were used where appropriate. Age was adjusted by univariate logistic regression analysis to reduce bias. A *p* value less than 0.05 was considered as statistically significant.

## Results

### Characteristics of Study Participants

A total of 45 participants were enrolled in the study, including 15 VKH subjects (male/female: 8/7), 15 BD subjects (male/female: 9/6), and 15 senile cataract controls (male/female: 6/9) (**Table 1**). No significant differences were present with regards to sex and IOP among the groups (*p* = 0.54 and *p* = 0.13, respectively). All the 30 uveitis patients were on treatment with systemic corticosteroids, without additional biologic agents. The number of VKH patients receiving immunosuppressants (15/15, 100%) was similar to that in the BD group (13/15, 86.57%). Overall, the treatment regimens did not vary between the BD and VKH groups.

### Aqueous Metabolomics in VKH, BD, and Controls

**Figure 1:**
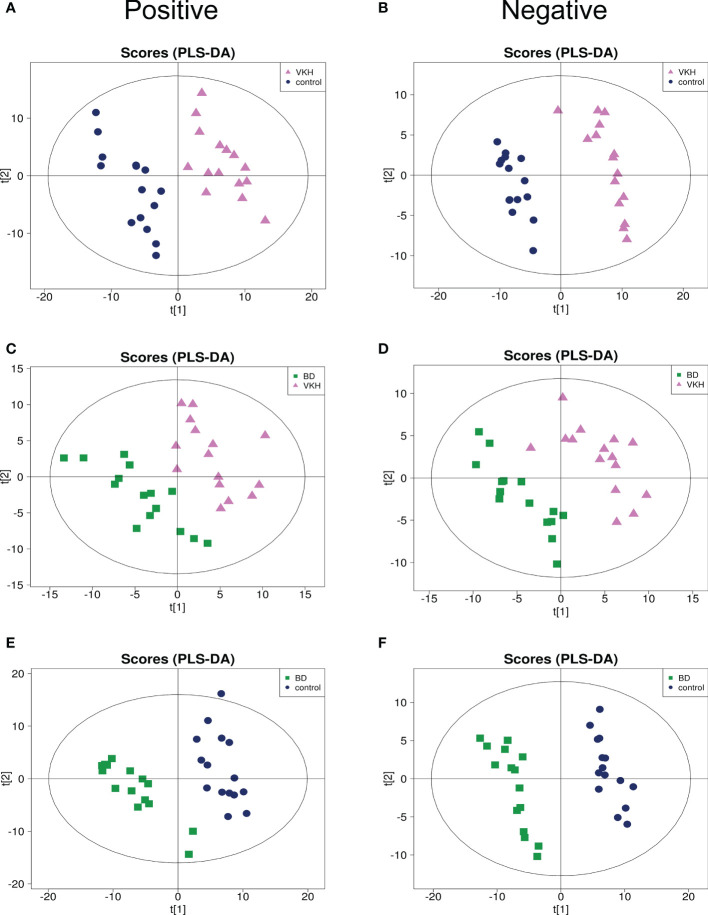
Partial least square discriminant analysis (PLS-DA) for any two of the three groups. PLS-DA plots for Vogt-Koyanagi-Harada disease (VKH) versus senile cataract controls separation in positive mode **(A)** and negative mode **(B)**; PLS-DA plots for VKH disease and Behcet’s disease (BD) separation in positive mode **(C)** and negative mode **(D)**; PLS-DA plots for BD and senile cataract controls separation in positive mode **(E)** and negative mode **(F)**.

To identify the aqueous metabolic profiles among the three groups tested, aqueous humor specimens were applied for untargeted metabolomics analysis. The total ion chromatograms are shown in **Figure S1**. Only slight changes of the spectral peaks of the QC samples were found, indicating that the method showed a satisfactory reproducibility. A total of 14,592 positive-model signals and 10,985 negative-model signals were detected in this study after signal filtering and peak alignment. After pareto scaling of the data, PCA models showed that the QC samples were tightly clustered in both positive and negative modes (**Figure S2**), which also suggests the high reproducibility of the method and credibility of the data. PLS-DA plots were performed to characterize the metabolic profiles for both positive and negative modes, and revealed clear differences when comparing VKH vs. control, BD vs. control, and VKH vs. BD (**Figure 1**).

### Differentially Expressed Metabolites Were Identified Between Groups

Metabolites with a VIP > 1 were filtered to perform Student’s t-test. The results were adjusted with FDR < 0.05 for multiple comparisons. To identify differentially expressed metabolites for subsequent analysis, we further raised the criteria to a VIP > 1.5. In total, 84 metabolites with a VIP > 1.5 and p < 0.05 (FDR corrected) were considered as differentially expressed between the groups (**Table S1**). HMDB was used to gain the classes of the 84 metabolites. It was found that amino acids and fatty acids were the two categories with the most abundant differential metabolites. As shown in **Figure S3**, 64 metabolites for VKH vs. control and 65 metabolites for BD vs. control showed significant differences. We found 27 differentially expressed metabolites between VKH and BD (data not shown). However, the significance was lost after FDR correction. The volcano plots were made to describe the variation tendencies of metabolites for VKH vs. control and BD vs. control (**Figure S4**).

**Figure 2:**
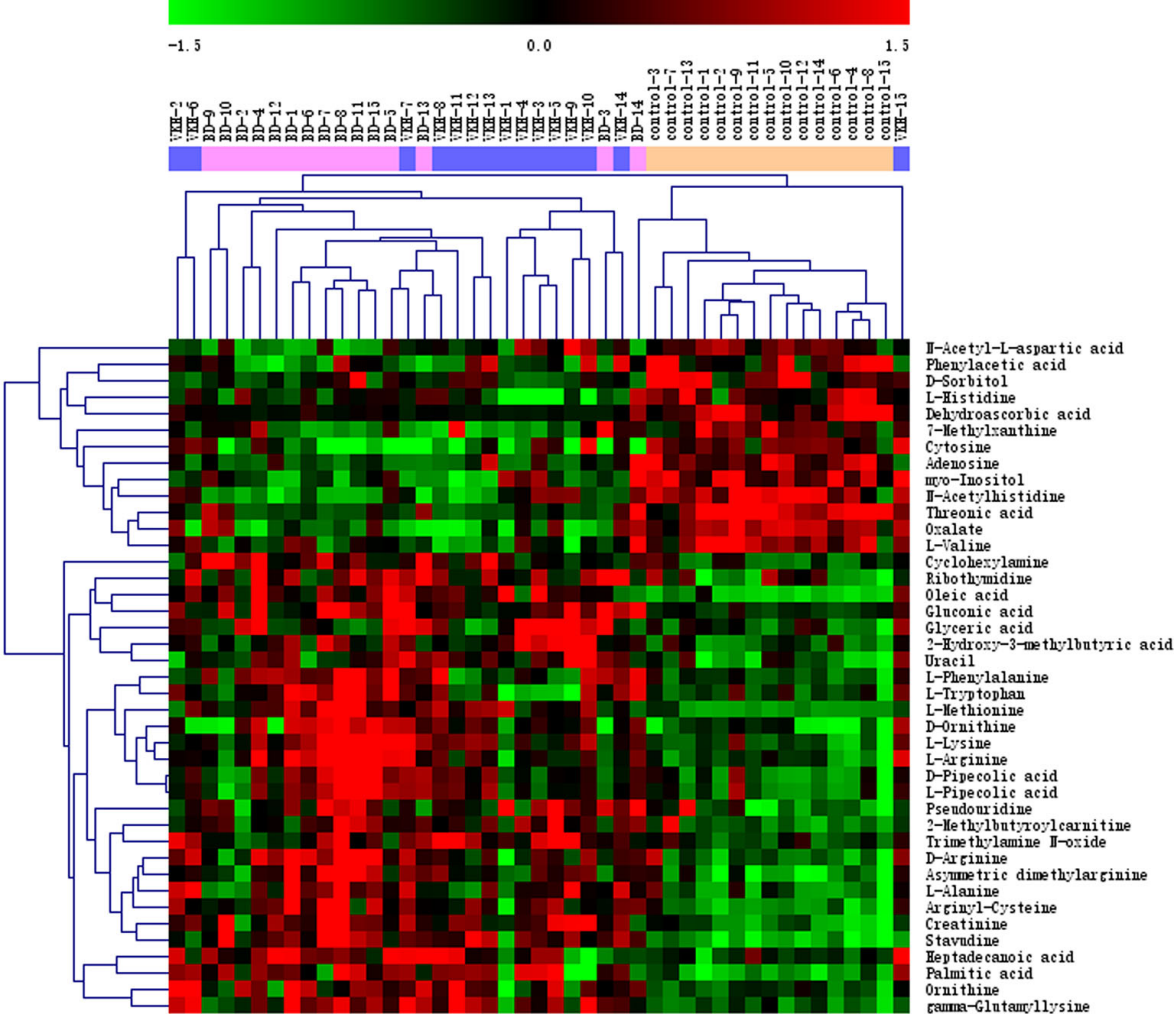
Heat plot of the significantly differential metabolites among three groups. VKH, Vogt-Koyanagi-Harada disease; BD, Behcet’s disease.

**Figure 3:**
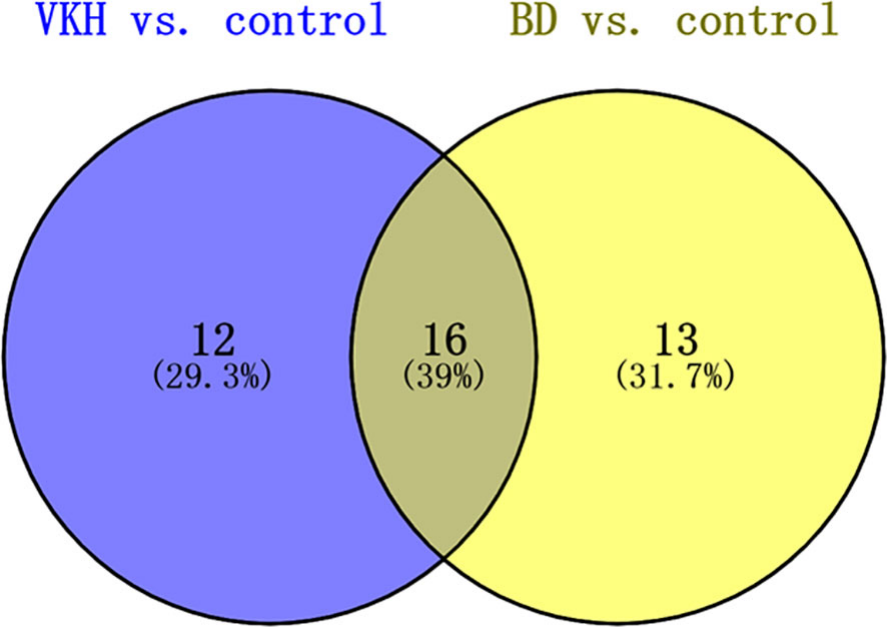
Venn diagram of metabolites after logistic regression analyses adjusting which significantly differed in comparisons between VKH disease, BD, and control groups. VKH, Vogt-Koyanagi-Harada disease; BD, Behcet’s disease.

To further investigate metabolites that could differentiate a group of patients from the other participants, we conducted univariate logistic regression analysis (**Table 2**). There were 41 differential metabolites between the groups after adjustment (**Figure 2**). There were 28 and 29 significantly differential metabolites for VKH vs. control and BD vs. control, respectively (**Figure 3**). Among them, nine amino acids and two fatty acids were overlapping differential metabolites. Palmitic acid and oleic acid levels, the two long-chain fatty acids, were significantly elevated in both VKH and BD compared to controls. Except for N-acetylhistidine, the other eight amino acids were significantly increased in both VKH and BD compared to controls, including asymmetric dimethylarginine, L-lysine, D-ornithine, D-pipecolic acid, L-methionine, creatinine, L-pipecolic acid, and gamma-Glutamyllysine. L-histidine, ornithine, and L-valine were differential amino acids when VKH patients were compared with controls. L-phenylalanine, D-arginine, N-acetyl-L-aspartic acid, L-alanine, arginyl-cysteine, and L-arginine were differential amino acids for BD vs. controls.

**Table 2:** Results of univariate logistic regression analysis comparing the differential metabolites levels of patients to those of controls.

Metabolites	VKH		BD	
	OR (95% CI)	*p* value	OR (95% CI)	*p* value
Pyroglutamic acid	–	–	–	–
L-Phenylalanine	–	–	13.054 (1.589–107.249)	**0.017**
Cyclohexylamine	–	–	63.537 (1.657–2,435.857)	**0.026**
Citramalic acid	–	–	5.975E+9 (0.334–1.068E+20)	0.062
L-Tyrosine	–	–	3.165 (0.728–13.761)	0.124
L-Tryptophan	–	–	14.504 (1.951–107.815)	**0.009**
gamma-Glutamylalanine	–	–	2.814 (0.42–18.854)	0.286
L-Glutamine	–	–	8.622 (0.891–83.397)	0.063
Arachidonic Acid	–	–	–	–
Uracil mustard	–	–	55.004 (0.216–14,034.571)	0.156
Pseudouridine	–	–	17.51 (1.043–293.893)	**0.047**
Phenylacetic acid	–	–	0.098 (0.015–0.652)	**0.016**
N-Alpha-acetyllysine	–	–	37.793 (0.81–1,764.335)	0.064
Heptadecanoic acid	–	–	5.641 (1.217–26.149)	**0.027**
D-Arginine	–	–	26.544 (2.55–276.276)	**0.006**
D-Lactic acid	–	–	0.366 (0.101–1.334)	0.128
Ribothymidine	–	–	9.92 (1.402–70.182)	**0.022**
Cytosine	–	–	0.198 (0.045–0.865)	**0.031**
N-Acetyl-L-aspartic acid	–	–	0.11 (0.019–0.644)	**0.014**
Androsterone sulfate	–	–	661.353 (0.142–3.087E+06)	0.132
Acetylcarnitine	218.881 (0.558–85,932.722)	0.077	–	–
Palmitic acid	5.908 (1.403–24.874)	**0.015**	5.137 (1.101–23.956)	**0.037**
Linoleic acid	1.096E+18 (0–3.648E+39)	0.1	3.69E+17 (0–1.223E+39)	0.11
Sorbitol	0.095 (0.011–0.843)	**0.035**	0.126 (0.014–1.146)	0.66
Asymmetric dimethylarginine	84.921 (1.512–4,769.689)	**0.031**	162.346 (2.59–10,176.585)	**0.016**
Betaine	2.906 (0.896–9.431)	0.076	–	–
Oleic acid	51.746 (2.053–1,304.351)	**0.017**	56.921 (2.086–1,553.162)	**0.017**
2-Methylbutyroylcarnitine	16.964 (1.481–194.372)	**0.023**	–	–
L-Histidine	0.121 (0.015–0.995)	**0.049**	–	–
D-Mannitol	0.425 (0.112–1.611)	0.208	–	–
Malonic acid	0.264 (0.067–1.041)	0.057	0.269 (0.055–1.303)	0.103
Glyceric acid	7.073 (1.128–44.344)	**0.037**	–	–
Uric acid	19.491 (0.552–687.894)	0.102	13.981 (0.385–507.987)	0.15
myo-Inositol	0.173 (0.036–0.824)	**0.028**	0.181 (0.034–0.948)	**0.043**
2-Hydroxy-3-methylbutyric acid	82.362 (1.693–4,007.462)	**0.026**	41.241 (0.851–1,997.65)	0.06
Threonic acid	0.02 (0.001–0.391)	**0.01**	0.028 (0.001–0.59)	**0.021**
Myristic acid	–	–	–	–
Trimethylamine N-oxide	182.074 (1.236–26,828.903)	**0.041**	–	–
N6-N6-N6-Trimethyl-L-lysine	1,706.774 (0.047–6.198E+07)	0.165	1,346.868 (0.037–4.916E+07)	0.179
Gamma-Linolenic acid	1.0042E+16 (0.046–2.349E+33)	0.07	3.922E+15 (0.018–8.718E+32)	0.078
Dehydroascorbic acid	4.264E−5 (4.047E−9–0.449)	**0.033**	0.001 (6.958E−7–1.951)	0.074
Urea	2.666 (0.802–8.861)	0.11	–	–
Coniferyl aldehyde	0.369 (0.108–1.266)	0.113	–	–
Hydroxyisocaproic acid	–	–	–	–
Palmitoleic acid	12.937 (0.581–288.155)	0.106	8.423 (0.37–191.537)	0.181
N-Acetylhistidine	0.147 (0.022–0.961)	**0.045**	0.038 (0.004–0.361)	**0.004**
L-Carnitine	1,067.081 (0.272–4.189E+06)	0.099	875.146 (0.222–3.443E+06)	0.109
L-Alanine	4.092 (0.823–20.35)	0.085	6.525 (1.095–38.886)	**0.039**
N-a-Acetyl-L-arginine	8.079 (0.677–96.481)	0.099	9.484 (0.748–120.32)	0.083
Melamine	0.349 (0.111–1.101)	0.072	–	–
L-Lysine	24.662 (1.365–445.649)	**0.03**	64.749 (3.039–1,379.643)	**0.008**
Oxalate	0.083 (0.01–0.708)	**0.023**	0.091 (0.01–0.854)	**0.036**
D-Proline	49,040.59 (0.371–6.479E+09)	0.073	22,919.578 (0.173–3.031E+09)	0.095
D-Galactarate	–	–	–	–
Citraconic acid	–	–	–	–
Xanthine	–	–	–	–
Allantoin	4.834 (0.652–35.834)	0.123	3.476 (0.44–27.484)	0.238
D-Ornithine	3.764 (1.026–13.807)	**0.046**	5.059 (1.182–21.652)	**0.029**
D-Pipecolic acid	13.908 (1.11–174.212)	**0.041**	28.391 (1.909–422.144)	**0.015**
L-Methionine	654.271 (1.167–366,750.445)	**0.045**	995.407 (1.702–582,209.079)	**0.034**
cis-Aconitic acid	–	–	–	–
Adenosine	0.097 (0.017–0.568)	**0.01**	–	–
3-Hydroxybutyric acid	0.4 (0.122–1.313)	0.131	0.379 (0.074–1.952)	0.246
Arginyl-Cysteine	245.905 (0.69–87,593.484)	0.066	538.096 (1.4–206,887.842)	**0.038**
L-Arginine	21.832 (0.923–516.491)	0.056	77.246 (2.72–2,193.341)	**0.011**
L-Cystine	–	–	–	–
Ornithine	225.963 (1.195–42,724.551)	**0.043**	–	–
Creatinine	273.817 (1.073–69,863.673)	**0.047**	357.107 (1.324–96,291.296)	**0.04**
3-Methoxy-4-Hydroxyphenylglycol Sulfate	56.764 (0.917–3,514.634)	0.055	31.913 (0.502–2,026.769)	0.102
Diethanolamine	0.049 (0.001–2.441)	0.13	0.026 (0–1.825)	0.093
L-Pipecolic acid	17.021 (1.272–227.676)	**0.032**	35.008 (2.225–550.864)	**0.011**
Gluconic acid	2,563.115 (2.053–3.2E+06)	**0.031**	6561.35 (4.931–8.731E+06)	**0.017**
Decanoylcarnitine	9,139.504 (0.401–2.084E+08)	0.075	–	–
Galacturonic acid	–	–	–	–
m-Chlorohippuric acid	2.328 (0.665–8.151)	0.186	2.558 (0.627–10.447)	0.191
Stearidonic acid	48083.35 (0.451–5.124E+09)	0.068	–	–
Uracil	56.065 (1.229–2557.482)	**0.039**	–	–
7-Methylxanthine	0.24 (0.059–0.984)	**0.047**	–	–
L-Valine	0.118 (0.022–0.643)	**0.013**	–	–
Methylmalonic acid	0.014 (2.655E−5–6.897)	0.176	0.017 (1.92E−05–14.773)	0.238
L-Malic acid	42,033.685 (0.085–2.076E+10)	0.111	–	–
Stavudine	162.849 (1.062–24,967.269)	**0.047**	272.228 (1.645–45,052.551)	**0.031**
gamma-Glutamyllysine	36.475 (1.135–1,171.916)	**0.042**	38.583 (1.147–1,297.405)	**0.042**
L-Kynurenine	24,202.338 (0.025–2.307E+10)	0.151	–	–

VKH, Vogt-Koyanagi-Harada disease; BD, Behcet’s disease; OR, odds ratio; CI, confidence interval.

P value in bold was less than 0.05 which was regarded as statistically significant.

### Pathway Analysis of Differential Aqueous Metabolites

**Figure 4:**
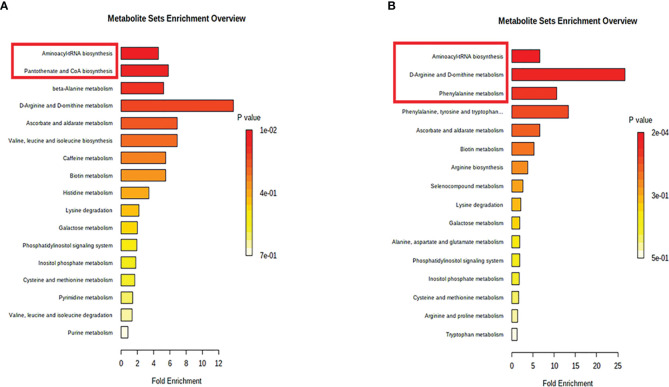
Pathway analysis of the differential metabolites in Vogt-Koyanagi-Harada disease group **(A)** and Behcet’s disease group **(B)**. The significantly perturbed pathways were in red rectangle.

Subsequently, MetaboAnalyst was used to compare metabolic disturbances between VKH, BD, and the control groups (**Figure 4**). There were two metabolic pathways that were significantly perturbed when comparing VKH patients with controls, and three pathways when comparing BD patients with controls (**Table 3**). The aminoacyl-tRNA biosynthesis pathway was significantly altered in both VKH and BD as compared to controls. In addition, we found significant differences in pantothenate and coenzyme A (CoA) biosynthesis for VKH vs. control, and in D-arginine and D-ornithine metabolism and phenylalanine metabolism for BD vs. control.

**Table 3:** The significantly altered pathways in Vogt-Koyanagi-Harada disease and Behcet’s disease.

Pathways	VKH			BD		
	*p* value	Hit	Metabolites	*p* value	Hit	Metabolites
Aminoacyl-tRNA biosynthesis	0.01	4	L-Histidine, L-Methionine, L-Lysine, L-Valine	<0.001	6	L-Phenylalanine, L-Arginine, L-Methionine, L-Alanine, L-Lysine, L-Tryptophan
D-Arginine and D-ornithine metabolism	–	–	–	0.002	2	D-Arginine, D-Ornithine
Phenylalanine metabolism	–	–	–	0.014	2	L-Phenylalanine, Phenylacetic acid
Pantothenate and CoA biosynthesis	0.045	2	L-Valine, Uracil	–	–	–

VKH, Vogt-Koyanagi-Harada disease; BD, Behcet’s disease.

## Discussion

In the present study, we found that aqueous humor from VKH and BD patients showed distinct metabolic profiles compared to senile cataract controls. After correction, 28 and 29 metabolites were differentially expressed for VKH vs. control and BD vs. control, respectively. Amino acids and fatty acids were the two most abundant differential metabolite categories. Also, significant alterations were found in several metabolic pathways, including aminoacyl-tRNA biosynthesis, pantothenate and CoA biosynthesis, D-arginine and D-ornithine metabolism and phenylalanine metabolism. Experimental verification is needed to validate the role of the pathways identified in this clinical study in the pathogenesis of uveitis. There are very good animal models of autoimmune uveitis (21–23) and further studies are planned to confirm our clinical findings in these models.

Fatty acids are of great importance for immune regulation and may contribute to the development of some autoimmune diseases (24–27). Previous studies have shown that serum oleic acid level and urine palmitic acid and oleic acid levels were significantly higher in BD (11, 28). Moreover, our recent studies in VKH disease identified that sweat palmitic acid level (10) and serum oleic acid level (unpublished data) were increased in VKH. In the current study aqueous palmitic acid and oleic acid levels were significantly higher in VKH and BD compared to controls. The elevated intraocular and peripheral levels of both palmitic acid and oleic acid levels observed in VKH and BD indicates that they may play an important role in the pathophysiology of uveitis. Further studies are needed to investigate the functions of these two fatty acids and their association in the pathogenesis of uveitis.

Amino acids are important protein building stones but can also play a role in the functioning of the immune system (29). It was reported that glutamine metabolism had a distinct role to promote Th17 cells but constrained Th1 cell differentiation (30). Moreover, alanine was recently found to be essential for early T cell activation and deprivation of it might impair T cell effector functions (31). Previous metabolic profiling of aqueous humor obtained from acute anterior uveitis and Posner–Schlossman syndrome patients showed differences concerning amino acid metabolism (32, 33). Our previous metabolomics study of sweat also revealed an important role for amino acid metabolism in the pathogenesis of VKH disease (10). In our present study, we found 12 and 15 amino acids were differentially expressed in VKH vs. control and BD vs. control, respectively. When compared to controls, D-arginine, D-ornithine, and L-phenylalanine, as well as its catabolite phenylacetic acid were significantly increased in BD patients, suggesting a possible role for the pathways regulating these specific amino acids in the pathogenesis of BD. Several studies have reported that the pantothenate and CoA biosynthesis pathway is associated with amino acid metabolism, whereby branched-chain amino acids provide CoA derivatives to enter the tricarboxylic acid cycle (29, 34, 35). The involvement of pantothenate and CoA biosynthesis was evident from a decrease in L-valine and increase in uracil that we observed in VKH compared to controls.

Another interesting finding from our current study came from the analysis of Aminoacyl-tRNAs, which are the essential substrates that transport specific amino acids to incorporate them into the polypeptide chain produced during translation (36, 37). The aminoacyl-tRNA biosynthesis pathway is associated with various diseases, including several autoimmune diseases (38–40). Of interest is our finding that the most significant pathway in our study was aminoacyl-tRNA biosynthesis which involved L-Histidine, L-Methionine, L-Lysine and L-Valine for VKH, and L-Phenylalanine, L-Arginine, L-Methionine, L-Alanine, L-Lysine and L-Tryptophan for BD. Both VKH disease and BD showed an increase of L-Methionine and L-Lysine and the involvement of the associated aminoacyl-tRNA biosynthesis pathway (**Figure 3**). Therefore, we speculated that these two uveitis entities might share metabolic regulation pathways. Although these two uveitis entities show very specific features, they could be driven by common immunopathological mechanisms (41, 42).

There are several limitations in the current study. First, for ethical reasons, we used aqueous humor samples obtained during cataract surgery. Prior to cataract surgery for uveitis, the inflammation in these eyes has to be controlled and patients enrolled in our study are therefore in an inactive state. Despite this limitation, we do believe that the data are interesting because the composition of the aqueous humor can reflect the status of the basic intraocular environment in a uveitis patient. Second, the sample size used in our study may seem small, but aqueous humor is not easily accessible like serum or urine. In view of ethical considerations and current practice in our hospital, we only collected aqueous humor when the patient was undergoing an operation to remove a cataract from their eyes. These operations are not very frequently done and it therefore took us quite some time to collect the desired number of samples from these two well-defined uveitis entities. Most aqueous metabolomics studies in the field of ophthalmology consist of less than 30 samples in each group (32, 33). The relatively small sample size might limit the finding of other differentially expressed metabolites. This may explain why the significance of differential metabolites was lost when comparing VKH with BD. Both entities are very different from each other, both from a clinical and a pathological perspective (6). A larger population is therefore needed to validate the results in future. Third, patients enrolled in our study were still on treatment with a low dose of systemic corticosteroids and immunosuppressants and we cannot exclude an effect of treatment on our findings. It is not ethical to terminate therapy in these patients for the mere purpose of research. On the other hand our data are different from those reported in an earlier study on glucocorticoid-induced metabolome changes (43), and are in agreement with metabolic studies in uveitis (10, 11). Our control group consisted of senile cataract patients whose age is inevitable older than our uveitis patients, but again, it is not ethically justified to collect aqueous samples from age matched healthy controls. In view of this limitation, we used a univariate logistic regression analysis to adjust for age. Using an age-stratified analysis we found no significant differences for the 41 differential metabolites between the different age groups (**Table S2**). In our study we set the accuracy m/z value at 25 ppm, although a more strict setting of 10 ppm might also have been used. Many previous reports in this field use a setting of 25 ppm (10, 44). We believe that several metabolites might have been missed if one would use a smaller accuracy m/z value.

In summary, we found specific aqueous metabolic profiles for VKH disease and BD. Differential amino acid and fatty acid expression in aqueous humor could play important roles in the pathogenesis of the intraocular inflammation in these two diseases. We further provide evidence for a possible role of the aminoacyl-tRNA biosynthesis pathway in uveitis. Further research is needed to investigate the exact role of these metabolites and relevant metabolic pathways in the diagnosis and treatment of VKH disease and BD.

## Data Availability Statement

The original contributions presented in the study are included in the article/**Supplementary Material**. Further inquiries can be directed to the corresponding author.

## Ethics Statement

The studies involving human participants were reviewed and approved by the Ethics Committee of the First Affiliated Hospital of Chongqing Medical University. The patients/participants provided their written informed consent to participate in this study.

## Author Contributions

PY and JX conceived the idea and designed the study. PY, JX, and QC contributed to collecting the aqueous humor and clinical data. JX, XH, RC, ZC, ZY, and GS performed the experiments and analyzed the data. JX wrote the manuscript. PY and AK reviewed data interpretation and edited the manuscript. All authors contributed to the article and approved the submitted version.

## Funding

This study was supported by National Natural Science Foundation Key Program (81930023), Natural Science Foundation Major International (Regional) Joint Research Project (81720108009), Chongqing Outstanding Scientists Project (2019), Chongqing Key Laboratory of Ophthalmology (CSTC, 2008CA5003), Chongqing Science & Technology Platform and Base Construction Program (cstc2014pt-sy10002), and the Chongqing Chief Medical Scientist Project (2018).

## Conflict of Interest

The authors declare that the research was conducted in the absence of any commercial or financial relationships that could be construed as a potential conflict of interest.
