# Supplementary material for: Metabolomic Analysis of Aqueous Humor Identifies Aberrant Amino Acid and Fatty Acid Metabolism in Vogt-Koyanagi-Harada and Behcet’s Disease
Source: Front Immunol. 2021 Feb 22;12:587393. doi: 10.3389/fimmu.2021.587393 (PMC7959366; doi:10.3389/fimmu.2021.587393)
Supplement: Supplementary file 1 [file DataSheet_1.doc]

**Figure S1.** The total ion chromatograms of the quality control samples in both positive model (A) and negative model (B).

**
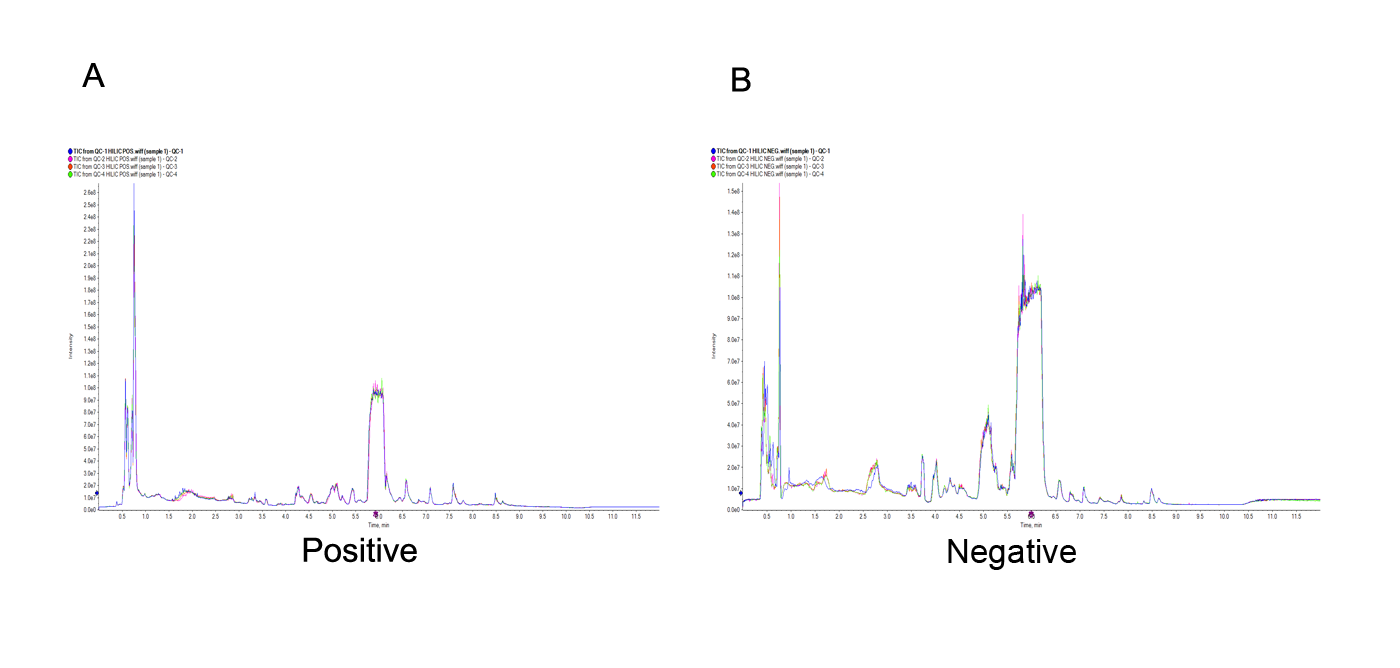
**

**Figure S2.** Principal component analysis (PCA) plots based on the UHPLC-Q-TOF/MS data of aqueous humor samples in both positive model (A) and negative model (B).


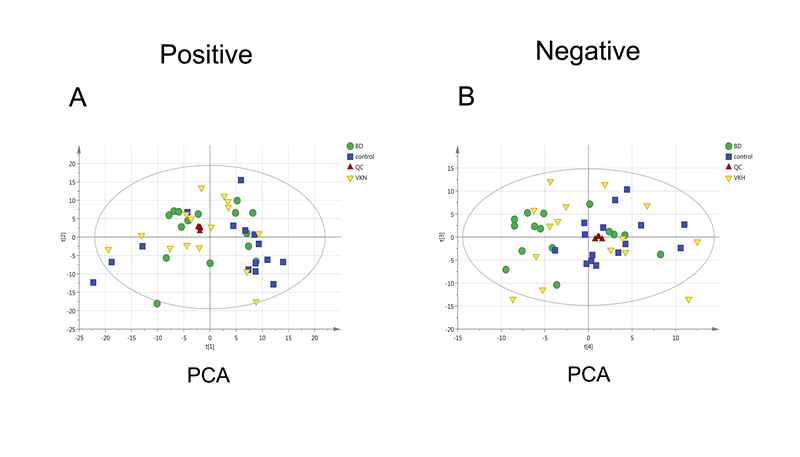


VKH, Vogt-Koyanagi-Harada disease; BD, Behcet’s disease; QC, quality control.

**Figure S3.** Venn diagram of aqueous metabolites which significantly differed in comparisons between VKH disease, BD and control groups


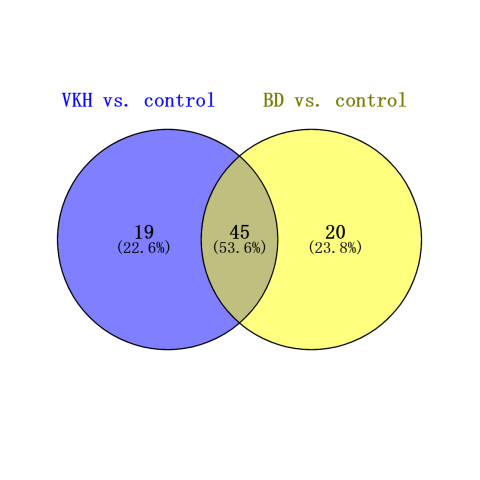


VKH, Vogt-Koyanagi-Harada disease; BD, Behcet’s disease.

**Figure S4.** Volcano plots of detected aqueous metabolites

**
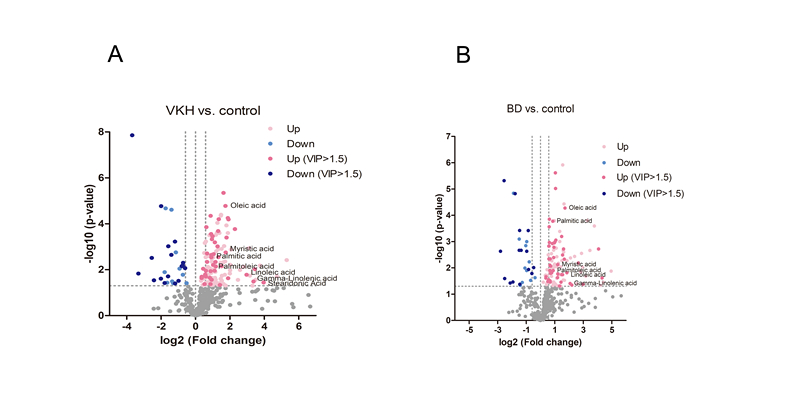
**

Volcano plots illustrated the association of metabolites with VKH disease (A) or BD (B), demonstrated as fold change (x-axis) and *p* value (y-axis).

The dashed line on y-axis indicated *p* value = 0.05.

The dashed lines on x-axis indicated fold change =0.67 (left) and 1.5 (right).

VKH, Vogt-Koyanagi-Harada disease; BD, Behcet’s disease.

Table S1. Differential metabolites were identified among the 3 groups.

| **Metabolites** | **VKH vs control** | | | **BD vs control** | | | **ESI+/−** | **Rt (sec)** | **m/z** | **Classes** |
| --- | --- | --- | --- | --- | --- | --- | --- | --- | --- | --- |
|  | **VIP** | **FC** | ***p* value** | **VIP** | **FC** | ***p* value** |  |  |  |  |
| Diethanolamine | 2.12 | 0.25 | 0.025 | 1.80 | 0.26 | 0.034 | + | 306.46 | 106.09 | Amines |
| Pyroglutamic acid | / | / | / | 19.70 | 2.08 | <0.001 | - | 303.48 | 128.04 | Amino acids |
| L-Phenylalanine | / | / | / | 9.94 | 1.54 | <0.001 | - | 256.54 | 164.07 | Amino acids |
| L-Tyrosine | / | / | / | 4.31 | 1.30 | 0.024 | - | 300.75 | 180.07 | Amino acids |
| gamma-Glutamylalanine | / | / | / | 2.37 | 2.53 | 0.047 | - | 348.54 | 199.07 | Amino acids |
| L-Glutamine | 1.39 | 1.42 | 0.024 | 2.35 | 1.52 | <0.001 | - | 396.6 | 145.06 | Amino acids |
| N-Alpha-acetyllysine | 1.43 | 2.33 | 0.037 | 1.98 | 2.29 | 0.023 | + | 429.46 | 249.14 | Amino acids |
| D-Arginine | / | / | / | 1.80 | 1.61 | 0.005 | + | 509.92 | 219.08 | Amino acids |
| N-Acetyl-L-aspartic acid | / | / | / | 1.69 | 0.55 | <0.001 | - | 392.97 | 174.04 | Amino acids |
| Asymmetric dimethylarginine | 9.95 | 1.46 | <0.001 | 13.51 | 1.59 | 0.005 | + | 490.03 | 203.15 | Amino acids |
| L-Histidine | 8.00 | 0.66 | 0.009 | / | / | / | + | 374.56 | 156.08 | Amino acids |
| N6,N6,N6-Trimethyl-L-lysine | / | / | / | 4.15 | 1.59 | 0.031 | + | 524.23 | 189.16 | Amino acids |
| N-Acetylhistidine | 3.78 | 0.57 | 0.007 | 5.34 | 0.29 | <0.001 | - | 317.94 | 196.07 | Amino acids |
| L-Alanine | 3.46 | 1.52 | 0.011 | 3.23 | 1.54 | 0.001 | + | 347.85 | 88.04 | Amino acids |
| N-a-Acetyl-L-arginine | 3.40 | 2.68 | 0.047 | / | / | / | + | 415.95 | 277.15 | Amino acids |
| L-Lysine | 3.38 | 1.59 | 0.002 | 6.17 | 1.96 | 0.001 | - | 521.11 | 145.1 | Amino acids |
| D-Proline | 3.17 | 2.86 | 0.007 | 2.42 | 1.89 | 0.004 | - | 317.7 | 114.06 | Amino acids |
| D-Ornithine | 2.77 | 2.03 | 0.006 | 4.39 | 2.33 | 0.017 | - | 508.88 | 131.08 | Amino acids |
| D-Pipecolic acid | 2.75 | 1.28 | 0.02 | 5.24 | 1.49 | 0.028 | + | 518.96 | 130.09 | Amino acids |
| L-Methionine | 2.97 | 4.89 | <0.001 | 3.64 | 6.26 | 0.007 | - | 282.83 | 148.04 | Amino acids |
| Arginyl-Cysteine | 2.31 | 2.48 | <0.001 | 3.26 | 3.07 | 0.002 | + | 418.37 | 341.13 | Amino acids |
| L-Arginine | 2.25 | 1.48 | 0.016 | 5.47 | 2.02 | 0.001 | - | 512.79 | 173.1 | Amino acids |
| L-Cystine | 2.25 | 1.94 | <0.001 | 1.87 | 1.59 | 0.008 | + | 426.86 | 241.03 | Amino acids |
| Ornithine | 2.22 | 1.82 | 0.043 | 1.14 | 1.41 | 0.019 | + | 501.96 | 133.1 | Amino acids |
| Creatinine | 2.22 | 1.55 | <0.001 | 2.26 | 1.50 | 0.001 | - | 172.26 | 112.05 | Amino acids |
| L-Pipecolic acid | 2.08 | 1.28 | 0.023 | 4.27 | 1.49 | 0.03 | + | 518.93 | 147.11 | Amino acids |
| L-Valine | 1.72 | 0.58 | 0.005 | / | / | / | + | 264.88 | 159.11 | Amino acids |
| gamma-Glutamyllysine | 1.62 | 2.42 | <0.001 | 1.81 | 2.31 | 0.008 | + | 463.39 | 317.18 | Amino acids |
| Betaine | 9.65 | 1.36 | 0.009 | / | / | / | + | 271.57 | 118.09 | Amino acids |
| Trimethylamine N-oxide | 5.26 | 1.99 | <0.001 | / | / | / | + | 376.48 | 146.12 | Aminoxides |
| myo-Inositol | 6.70 | 0.61 | 0.005 | 6.20 | 0.66 | 0.016 | - | 395.21 | 179.06 | Alcohols and polyols |
| D-Lactic acid | / | / | / | 1.70 | 0.72 | 0.01 | - | 334.43 | 89.02 | Alpha hydroxy acids |
| Phenylacetic acid | / | / | / | 2.01 | 0.56 | 0.011 | + | 35.66 | 119.05 | Benzenoids |
| m-Chlorohippuric acid | 1.91 | 1.44 | 0.042 | 2.68 | 1.57 | 0.012 | - | 171.9 | 213.02 | Benzoic acids |
| 3-Hydroxybutyric acid | 2.33 | 0.17 | 0.003 | 2.28 | 0.14 | 0.002 | - | 397.28 | 103.04 | Beta hydroxy acids |
| L-Malic acid | 1.69 | 2.51 | 0.003 | 1.21 | 2.05 | 0.04 | - | 402.9 | 133.01 | Beta hydroxy acids |
| Threonic acid | 5.90 | 0.25 | <0.001 | 5.32 | 0.36 | <0.001 | - | 379.73 | 135.03 | Carbohydrates |
| D-Galactarate | 3.15 | 0.08 | <0.001 | 3.01 | 0.17 | <0.001 | - | 224.81 | 191.02 | Carbohydrates |
| Glyceric acid | 6.91 | 1.84 | 0.012 | / | / | / | - | 309.82 | 105.02 | Carbohydrates |
| Gluconic acid | 2.02 | 2.23 | 0.005 | 2.88 | 3.18 | 0.005 | - | 373.74 | 195.05 | Carbohydrates |
| Mannitol | 7.83 | 0.44 | 0.04 | / | / | / | + | 46.22 | 165.07 | Carbohydrates |
| Sorbitol | 10.32 | 0.33 | 0.02 | 8.66 | 0.37 | 0.042 | + | 77.56 | 165.08 | Carbohydrates |
| Galacturonic acid | 1.93 | 2.18 | <0.001 | 1.69 | 1.93 | 0.002 | - | 387.67 | 193.03 | Carbohydrates |
| L-Kynurenine | 1.58 | 10.94 | 0.01 | / | / | / | + | 306.87 | 209.09 | Carbonyl compounds |
| Cyclohexylamine | / | / | / | 6.92 | 2.83 | 0.018 | + | 383.44 | 160.13 | Cyclohexylamines |
| Malonic acid | 7.35 | 0.38 | 0.002 | 7.08 | 0.39 | 0.002 | - | 301.87 | 103 | Dicarboxylic acids |
| Oxalic acid | 3.25 | 0.44 | <0.001 | 2.97 | 0.51 | 0.001 | - | 413.47 | 88.99 | Dicarboxylic acids |
| Methylmalonic acid | 1.69 | 0.19 | 0.029 | 1.71 | 0.23 | 0.038 | - | 78.49 | 117.02 | Dicarboxylic acids |
| L-Acetylcarnitine | 14.34 | 1.51 | 0.02 | / | / | / | + | 304.32 | 204.12 | Fatty acid esters |
| 2-Methylbutyroylcarnitine | 8.71 | 1.78 | 0.023 | / | / | / | + | 239.44 | 246.17 | Fatty acid esters |
| Citramalic acid | / | / | / | 4.60 | 19.93 | 0.024 | - | 313.96 | 207.05 | Fatty acids |
| Arachidonic Acid | / | / | / | 2.34 | 16.81 | 0.002 | - | 42 | 303.23 | Fatty acids |
| Heptadecanoic acid | / | / | / | 1.88 | 1.63 | 0.009 | - | 43.53 | 269.25 | Fatty acids |
| Palmitic acid | 13.73 | 1.89 | 0.003 | 14.39 | 1.82 | <0.001 | - | 44.88 | 255.23 | Fatty acids |
| Oleic acid | 9.01 | 3.33 | <0.001 | 9.36 | 3.29 | <0.001 | - | 102.32 | 281.25 | Fatty acids |
| 2-Hydroxy-3-methylbutyric acid | 6.25 | 2.26 | 0.007 | 4.08 | 1.60 | 0.034 | - | 146.8 | 117.06 | Fatty acids |
| Myristic acid | 5.53 | 3.37 | 0.002 | 4.38 | 2.33 | 0.007 | - | 45.44 | 227.2 | Fatty acids |
| Hydroxyisocaproic acid | 3.89 | 3.09 | <0.001 | 3.57 | 2.79 | <0.001 | - | 119.11 | 131.07 | Fatty acids |
| Palmitoleic acid | 3.86 | 2.16 | 0.011 | 3.36 | 1.85 | 0.013 | - | 44.15 | 253.22 | Fatty acids |
| Citraconic acid | 2.82 | 3.67 | <0.001 | 2.18 | 2.81 | 0.018 | - | 438.08 | 129.02 | Fatty acids |
| Decanoylcarnitine | 1.98 | 3.72 | 0.024 | / | / | / | + | 162.66 | 316.25 | Fatty acids |
| Dehydroascorbic acid | 4.61 | 0.10 | 0.015 | 4.61 | 0.18 | 0.025 | - | 153.14 | 173.01 | Gamma butyrolactones |
| Allantoin | 2.78 | 1.89 | 0.002 | 3.03 | 1.79 | 0.001 | - | 290.32 | 139.03 | Imidazoles |
| L-Tryptophan | / | / | / | 3.91 | 1.50 | 0.005 | - | 257.74 | 203.08 | Indoles |
| Linoleic acid | 12.64 | 7.84 | 0.017 | 7.89 | 3.60 | 0.015 | - | 43.75 | 279.23 | Lineolic acids |
| Gamma-Linolenic acid | 5.05 | 10.37 | 0.033 | 2.92 | 4.22 | 0.039 | - | 44.34 | 277.22 | Lineolic acids |
| Stearidonic acid | 1.90 | 15.51 | 0.036 | / | / | / | - | 45.11 | 275.2 | Lineolic acids |
| 3-Methoxy-4-Hydroxyphenylglycol Sulfate | 2.18 | 2.07 | 0.002 | 1.69 | 1.64 | 0.016 | - | 42.25 | 263.02 | Methoxyphenols |
| Uracil mustard | / | / | / | 2.29 | 4.59 | 0.045 | - | 39.44 | 250.02 | Nitrogen mustard compounds |
| Stavudine | 1.63 | 1.83 | <0.001 | 2.26 | 2.06 | <0.001 | - | 388.08 | 224.08 | Nucleosides |
| Ribothymidine | 1.11 | 1.33 | 0.047 | 1.69 | 1.43 | 0.033 | - | 145.54 | 257.08 | Nucleosides |
| Adenosine | 2.35 | 0.33 | <0.001 | / | / | / | + | 170.21 | 268.1 | Nucleosides |
| Pseudouridine | 1.38 | 1.26 | 0.031 | 2.05 | 1.31 | 0.022 | - | 246.81 | 243.06 | Nucleosides |
| Coniferyl aldehyde | 3.91 | 0.60 | 0.006 | / | / | / | + | 390.89 | 178.06 | Other |
| Melamine | 3.40 | 0.29 | 0.038 | / | / | / | + | 178.8 | 127.07 | Other |
| Uric acid | 6.90 | 3.48 | <0.001 | 6.04 | 2.93 | 0.003 | - | 331.28 | 167.02 | Purines |
| Xanthine | 2.78 | 2.55 | <0.001 | 2.48 | 2.11 | <0.001 | - | 215.01 | 151.03 | Purines |
| 7-Methylxanthine | 1.75 | 0.50 | 0.031 | / | / | / | - | 450.15 | 165.04 | Purines |
| Cytosine | / | / | / | 1.69 | 0.36 | <0.001 | + | 239.31 | 112.05 | Pyrimidines |
| Uracil | 1.79 | 1.60 | 0.005 | 1.46 | 1.38 | 0.012 | - | 166.08 | 111.02 | Pyrimidines |
| L-Carnitine | 3.52 | 1.87 | <0.001 | 3.38 | 1.72 | 0.015 | + | 379.19 | 162.11 | Quaternary ammonium salts |
| Androsterone sulfate | 1.27 | 6.02 | 0.01 | 1.59 | 7.86 | 0.042 | - | 29.38 | 369.17 | Sulfated steroids |
| cis-Aconitic acid | 2.43 | 3.78 | <0.001 | 1.91 | 2.93 | 0.018 | - | 438.07 | 173.01 | Tricarboxylic acids |
| Urea | 4.38 | 1.91 | 0.028 | / | / | / | + | 94.55 | 61.04 | Ureas |

VKH, Vogt-Koyanagi-Harada disease; BD, Behcet’s disease; VIP, variable importance in the projection; FC, fold change; ESI, electrospray ionization; Rt, retention time.

P value was calculated by Student’s t-test adjusted with the critical false discovery rate set to 0.05 for multiple comparisons.

Table S2. Results of age-stratified analysis comparing the differential metabolites levels.

| **Metabolites** | **Controls** | | ***p* value** |
| --- | --- | --- | --- |
|  | **age ≤ 60 (n=6)** | **age > 60 (n=9)** |  |
| L-Phenylalanine | 3260044.01 ± 389065.7 | 2996436.05 ± 445719.49 | 0.937 |
| Cyclohexylamine | 445574.55 ± 327164.66 | 368551.19 ± 240621.2 | 0.939 |
| L-Tryptophan | 640959.2 ± 79894.91 | 587041.37 ± 145433.81 | 0.937 |
| Pseudouridine | 431452.04 ± 27479.6 | 393355.32 ± 130919.62 | 0.939 |
| Phenylacetic acid | 116808.72 ± 30577.06 | 120476.44 ± 51858.59 | 0.939 |
| Heptadecanoic acid | 103089.1 ± 33254.18 | 133254.12 ± 27765.7 | 0.810 |
| D-Arginine | 62950.25 ± 20591.93 | 55294.77 ± 14206.76 | 0.937 |
| Ribothymidine | 150961.21 ± 59731.23 | 140360.47 ± 51537.08 | 0.939 |
| Cytosine | 66470.58 ± 7003.54 | 54170.61 ± 13808.99 | 0.810 |
| N-Acetyl-L-aspartic acid | 81588.52 ± 12232.12 | 66700.13 ± 13351.88 | 0.810 |
| Palmitic acid | 3504584.74 ± 1154054.07 | 4071516.35 ± 933884.93 | 0.937 |
| Asymmetric dimethylarginine | 3332967.45 ± 897363.56 | 3337935.32 ± 1057260.75 | 0.993 |
| Oleic acid | 647085.99 ± 419977.73 | 487631.83 ± 205860.27 | 0.937 |
| myo-Inositol | 1428145.19 ± 257919.51 | 1321857.07 ± 360983.94 | 0.939 |
| Threonic acid | 455894.95 ± 188235.37 | 509944.05 ± 198233.19 | 0.939 |
| N-Acetylhistidine | 461924.06 ± 94684.35 | 441759.38 ± 170171.53 | 0.939 |
| L-Alanine | 80771.76 ± 76942.35 | 51063.88 ± 60639.63 | 0.937 |
| L-Lysine | 668579.82 ± 137339.25 | 600006.94 ± 247743.23 | 0.939 |
| Oxalate | 205334.97 ± 32598.19 | 199097.02 ± 49438.46 | 0.939 |
| D-Ornithine | 232014.22 ± 205542.57 | 273104.1 ± 181510.56 | 0.939 |
| D-Pipecolic acid | 778190.95 ± 160343.58 | 785581.54 ± 242246.26 | 0.973 |
| L-Methionine | 22973.53 ± 24145.98 | 24658.34 ± 18133 | 0.939 |
| Arginyl-Cysteine | 64618.85 ± 35446.61 | 45183.88 ± 21560.25 | 0.937 |
| L-Arginine | 484042.88 ± 98513.45 | 405682.58 ± 150401.71 | 0.937 |
| Creatinine | 180036.6 ± 28053.68 | 163436.5 ± 39600.62 | 0.937 |
| L-Pipecolic acid | 531517.41 ± 81573.45 | 560426.15 ± 175036.75 | 0.939 |
| Gluconic acid | 60232.42 ± 13417.88 | 82239.49 ± 25806.94 | 0.81 |
| Stavudine | 61139.47 ± 12287.34 | 54832.35 ± 15343.39 | 0.937 |
| gamma-Glutamyllysine | 26588.5 ± 9880.63 | 27374.95 ± 11502.71 | 0.939 |
| D-Sorbitol | 2221624.99 ± 1876761.51 | 2057629.74 ± 1285713.88 | 0.939 |
| 2-Methylbutyroylcarnitine | 1416770.91 ± 432879.46 | 1578823.88 ± 1131536.56 | 0.939 |
| L-Histidine | 1713983.77 ± 332170.05 | 1963518.52 ± 335382.6 | 0.937 |
| Glyceric acid | 1674541.79 ± 475394.64 | 1324498.44 ± 654831.87 | 0.937 |
| 2-Hydroxy-3-methylbutyric acid | 580123.12 ± 309449.47 | 621717.44 ± 282806.99 | 0.939 |
| Trimethylamine N-oxide | 337759.23 ± 92599.78 | 395942.74 ± 111058.7 | 0.937 |
| Dehydroascorbic acid | 219363.11 ± 177025.24 | 432594.59 ± 423480.44 | 0.937 |
| Adenosine | 81392.45 ± 30745.98 | 87779.55 ± 34493.54 | 0.939 |
| Ornithine | 232014.22 ± 205542.57 | 273104.1 ± 181510.56 | 0.939 |
| Uracil | 122712.19 ± 39981.96 | 118108.78 ± 39325.24 | 0.939 |
| 7-Methylxanthine | 80969.95 ± 32464.12 | 86258.8 ± 53098.25 | 0.939 |
| L-Valine | 102272.89 ± 31916.6 | 89503.22 ± 28781.98 | 0.937 |

Relative quantitative values of the differential metabolites are expressed as mean±standard deviation.

P value was calculated by Student’s t-test adjusted with the critical false discovery rate set to 0.05 for multiple comparisons.
